# A Hybrid Scheme of MCS Selection and Spectrum Allocation for URLLC Traffic under Delay and Reliability Constraints

**DOI:** 10.3390/e24050727

**Published:** 2022-05-20

**Authors:** Yuehong Gao, Haotian Yang, Xiao Hong, Lu Chen

**Affiliations:** School of Information and Communication Engineering, Beijing University of Posts and Telecommunications, Beijing 100876, China; haotiany@bupt.edu.cn (H.Y.); xiaohong@bupt.edu.cn (X.H.); chenluu@bupt.edu.cn (L.C.)

**Keywords:** URLLC, MCS selection, spectrum allocation, network calculus, data duplication

## Abstract

The Ultra-Reliable Low-Latency Communication (URLLC) is expected to be an important feature of 5G and beyond networks. Supporting URLLC in a resource-efficient manner demands optimal Modulation and Coding Scheme (MCS) selection and spectrum allocation. This paper presents a study on MCS selection and spectrum allocation to support URLLC. The essential idea is to establish an analytical connection between the delay and reliability requirements of URLLC data transmission and the underlying MCS selection and spectrum allocation. In particular, the connection factors in fundamental aspects of wireless data communication include channel quality, coding and modulation, spectrum allocation and data traffic characteristics. With this connection, MCS selection and spectrum allocation can be efficiently performed based on the delay and reliability requirements of URLLC. Theoretical results in the scenario of a 5G New Radio system are presented, where the Signal-to-Noise Ratio (SNR) thresholds for adaptive MCS selection, data-transmission rate and delay, as well as spectrum allocation under different configurations, including data duplication, are discussed. Simulation results are also obtained and compared with the theoretical results, which validate the analysis and its efficiency.

## 1. Introduction

Ultra-Reliable Low-Latency Communication (URLLC) is expected to be an important feature of 5G and beyond [1,2,3]. Typically, the data payload of URLLC is small but the latency or delay and reliability requirements are stringent. For instance, in an example given by 3GPP [1], the data packet has a size of 32 bytes, and the reliability and latency requirements are that the packet is transmitted and received at the user/application plane within 1 ms with success rate $1-10^{-5}$. In fact, there is a broad range of scenarios where even more stringent latency and reliability guarantees are needed [3].

In the literature, a lot of results, covering different aspects of URLLC, have emerged. Excellent reviews, e.g., on physical layer [4], coding [5], access control [6] and network level [7] and journal special issues are available, e.g., [8]. However, the existing results, when treating the reliability requirement, take the error contribution due to finite length coding [4,5] and the delay violation probability due to queueing [6,7,8] separately or indeed often ignoring the other part. In addition, few of these results take into account the impact of data duplication, which is an important technique proposed for URLLC to increase packet success rate while keeping the latency low [9].

Furthermore, a crucial aspect for URLLC to be implemented in real systems has surprisingly little been touched, which is the selection of a modulation and coding scheme (MCS). To the best of our knowledge, refs. [10,11] are the only attempts investigating how MCS selection should be considered in URLLC. More specifically, the authors of [10,11] proposed to measure the error probability of packet transmission and accordingly adapt the select of MCS. A fundamental limitation of such an approach is that, with a targeted error probability of $10^{-5}$ or lower, for the measurement to be meaningful, it must measure at least 1 million packets. Unfortunately, this implies the measurement time span will be in the order of minutes or hours (e.g., 1 packet per 1 ms) or even longer, when the channel condition may have changed significantly. This motivates us to take on a different approach where the error probability can be directly estimated from channel quality measures, e.g., Signal-to-Noise Ratio (SNR) that can be easily obtained.

The objective of this paper is to, through analysis, reveal a connection between the URLLC (k,d,ϵ)-tuple, where *k* denotes the data packet length, *d* the latency requirement and ϵ the reliability requirement and channel modulation, coding length and quality. In addition, this connection is extended to consider data duplication in the transmission. This connection is finally expressed via inequations, which can be used in many aspects, such as to estimate the maximum delay, to allocate minimum bandwidth and to find the most suitable modulation and coding scheme. In this work, the results of optimal MCS selection and spectrum allocation for URLLC are presented and discussed. These results will not only be useful for MCS selection and spectrum allocation, but also add to the literature, providing additional insights into quantifying and understanding the fundamental limits of URLLC.

Specifically, we focus our study on a system with block fading channel using Quadrature Amplitude Modulation (QAM), where the coding length effect, due to small data packet size and low latency requirement, cannot be ignored. Our contributions are several-fold. First, by extending the channel dispersion results [12,13] to the QAM channel, the error probability due to finite block length coding is estimated.

Second, queueing analysis is conducted based on the network calculus theory particularly the stochastic branch [14], which has been identified as an important analytical tool for URLLC [7], to reveal the relation between delay requirement and its violation probability. Third, the effect of using data duplication for URLLC is investigated. Based on these results jointly, QAM selection and spectrum allocation are suggested. Finally, numerical results under a 5G New Radio (NR) setting are presented and discussed. Simulation results are also obtained and compared with the theoretical results, which validate the analysis and its efficiency.

The rest is organized as follows. Section 2 describes the considered system model. Section 3 introduces the analysis from different aspects. Numerical and simulation results, which demonstrate and verify the analysis, are presented in Section 4. Finally, our concluding remarks and discussion are made in Section 5.

## 2. System Model

**Figure 1:**
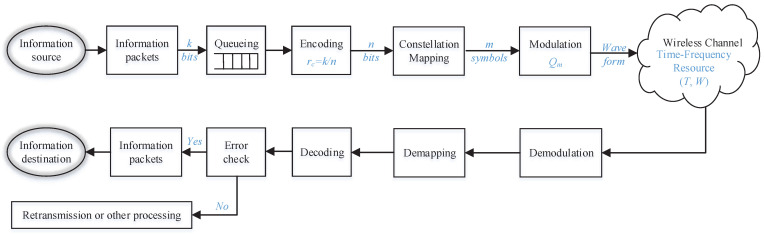
System Model.

A typical wireless link from the sender to the receiver is described in Figure 1, which includes all necessary modules to be used for the analysis in this work. The transmission starts from the information source, who generates information packets. As suggested in [15], the arrival of URLLC information packets is usually assumed to follow periodical model with fixed length. Here, let *k* denote the length of information packet. Then, an information packet will be transmitted out through one link or be duplicated and transmitted through multiple links. Data duplication, instead of Automatic Repeat Request (ARQ), is preferred for URLLC traffic to meet the reliability requirement, because ARQ requires retransmission after feedback from packet error detection at the receiver causing higher delay. Therefore, data duplication has been proposed for use in 5G NR system and beyond to achieve low latency while ensuring high packet success probability.

In each transmission link, the information packet will first goes through a queueing module, where it may go out directly when the system is idle or it may wait for some time if the system is busy. In this module, packet length will not be changed. Next, in the coding module, redundancy bits are added into the information packet in order to compensate for possible errors. Let *k* denote the length of a URLLC information packet in unit of bit and $r_{c}$ denote the coding rate, the length of the coded symbols (denoted by *n*) will be

(1) $$\begin{array}{c} n=\frac{k}{r_{c}}. \end{array}$$

After modulation, time-frequency resources are allocated for transmission through the wireless channel. The amount of allocated resources and channel quality will greatly impact the success probability and delay. At the receiver side, reverse operations are conducted to recover the information packet. When duplication transmission is applied, the information packet will be received correctly as far as one of the duplications is received correctly. In the next section, the delay and reliability of these URLLC packets going through the system described in Figure 1 will be analyzed.

## 3. The Analysis

### 3.1. Estimating the Error Probability

First, we consider any transmission link among several duplications. It has been proven that, if the generated information bits (with length of *k* bits) is first, encoded into a codeword with length of *n* symbols and then transmitted through a wireless channel at SNR of *P*, the channel transmission rate is upper bounded by Shannon capacity as

(2) $$C\left(P\right)=\log_{2}\left(1+P\right).$$

Note that, the upper bound given by Equation (2) requires Gaussian coding and infinite coding length (i.e., n→∞). Therefore, when the coding length is finite, it is not possible to realize reliable transmission with arbitrarily small error probability. In [12,13], the maximal rate achievable with error probability ϵ and finite coding length *n* is approximated. For the wireless channel, its equivalent baseband model is a complex Gaussian channel, which is composed of two real Additive White Gaussian Noise (AWGN) channels. Then, based on Theorem 4 in [12], the transmission rate under given error probability ϵ, and the finite coding length *n* is upper bounded by

(3) $$R_{1}\left(n,P,\epsilon \right)=C\left(P\right)-\sqrt{\frac{V(P)}{n}}Q^{-1}\left(\epsilon \right)+\frac{\log_{2}\left(n\right)}{n},$$

where

(4) $$\begin{array}{cc} Q(x) & =\int_{x}^{\infty }\frac{1}{\sqrt{2\pi }}e^{-t^{2}/2}dt,\\ V(P) & =\frac{P(P+2)}{{\left(P+1\right)}^{2}\ln^{2}2}. \end{array}$$

One important assumption in Equation (3) is Gaussian coding. However, in practical systems, a constellation diagram with limited points is usually used, such as the widely used M-QAM modulation. Then, the maximal rate under infinite coding length M-QAM modulation is no longer C(P) defined in Equation (2), the following upper bound should be used instead

(5) $$I\left(P,M\right)=\log_{2}M-\frac{1}{M\pi }\sum_{i=1}^{M}\int e^{-∥y-\sqrt{P}x_{i}{∥}^{2}}\times \left(\sum_{k=1}^{M}e^{-|y-\sqrt{P}x_{i}{|}^{2}-{\left|y-\sqrt{P}x_{k}\right|}^{2}}\right)dy.$$

However, there is no closed form for Equation (5). Fortunately, the authors of [16] found an approximation based on multi-exponential delay curve fitting (M-EDCF) as

(6) $$I^{\prime }\left(P,M\right)\approx \log_{2}M\times \left(1-\sum_{j=1}^{k_{M}}a_{j}^{\left(M\right)}e^{-b_{j}^{\left(M\right)}P}\right),$$

where the fitting coefficients for M-QAM are summarized in Table 1.

Then, for finite coding length and finite constellation, the maximal achievable transmission rate can be approximated as

(7) $$R_{2}\left(n,P,\epsilon ,M\right)=I^{\prime }\left(P,M\right)-\sqrt{\frac{V(P)}{n}}Q^{-1}\left(\epsilon \right)+\frac{\log_{2}\left(n\right)}{n}.$$

Based on the analysis above, the packet error probability for a single link can be derived by the following lemma.

**Lemma** **1.** *Consider an information packet with length of k bits transmitted via a wireless link under channel condition P and transmission rate $R_{2}$ using coding and modulation scheme with n and M, the packet error probability can be obtained by*

(8) $$\begin{array}{c} \epsilon \left(n,P,M\right)=Q\left(\sqrt{\frac{n}{V(P)}}\left(I^{\prime }\left(P,M\right)-R_{2}+\frac{\log_{2}\left(n\right)}{n}\right)\right). \end{array}$$

Note that, in the analysis of this section, the wireless channel is supposed to be a block fading channel, which means the fading in different time-frequency resource is the same for all *n* symbols in a transmission block. When the fadings over different symbols are also different, we only need to update the expressions for channel mutual information, i.e., C(P) and $I^{\prime }\left(P,M\right)$, the analysis in the remaining part of this paper still holds.

### 3.2. Meeting the Delay Requirement

In order to analyze the delay and reliability guarantee of the aforementioned system, a suitable analysis model is needed. In this work, network calculus will be relied on to establish a corresponding mathematical model for the considered system.

**Figure 2:**
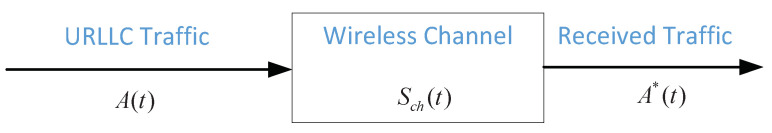
Equivalent Analysis Model for the Considered Wireless Communication System.

Network calculus is a queueing theory for QoS analysis [14], which has been widely used for performance evaluation in various networks. For the consider URLLC traffic model and wireless channel model described in Section 2, an equivalent analysis model for single link transmission can be abstracted as Figure 2 shows. The arrival process, denoted by A(t), is composed of all information packets generated from a URLLC information source; while the service process, denoted by $S_{ch}\left(t\right)$, is the service provided by the wireless channel. In network calculus analysis, arrival curve and service curve are defined to describe the characteristics of (cumulative) arrival process and (cumulative) service process, respectively, as follows.

**Definition** **1 (Arrival Curve).** *An arrival process A(t) is said to have an arrival curve α(t) if for all 0≤s≤t [14],*

(9) $$\begin{array}{c} A(t)-A(s)\leq \alpha (t-s), \end{array}$$

*or equivalently A(t−s)≤α(t−s), where A(t) is the amount of traffic arrived during period [0,t).*

**Definition** **2 (Service Curve).** Consider a system S with input process A(t) and output process $A^{*}\left(t\right)$. The system is said to have a service curve β(t) if for all t≥0 [14],

(10) $$\begin{array}{c} A^{*}\left(t\right)\geq A⊗\beta \left(t\right), \end{array}$$

*where “*⊗*” is the min-plus convolution, and $A⊗\beta \left(t\right)=\inf_{0\leq s\leq t}\left[A\left(s\right)+\beta \left(t-s\right)\right]$.*

For the considered URLLC traffic, it is assumed to be a periodical process with interval of τ and information bit length of *k*, then its arrival curve $\alpha_{u}\left(t\right)$ can be expressed as

(11) $$\alpha_{u}\left(t\right)=\frac{k}{\tau }t+k.$$

For the considered single wireless channel with finite coding length and finite constellation, when SNR *P*, coding rate $r_{c}$, error probability ϵ and modulation order *M* are given, and when the bandwidth *W* for transmission is allocated, the service process $S_{ch}\left(t\right)$ has service curve $\beta_{ch}\left(t\right)$ as:

(12) $$\beta_{ch}\left(t\right)=R_{2}\left(n,P,\epsilon ,M\right)\cdot W\cdot t.$$

Then, the delay of an information packet transmitted via a single link can be derived and summarized in Lemma 2. Detailed proof is given in Theorem 2.19 in [14].

**Lemma** **2.** *If the URLLC traffic follows a periodical process with an arrival curve of $\alpha_{u}\left(t\right)$, and it is transmitted through a wireless channel with a service curve of $\beta_{ch}\left(t\right)$, the delay D(t) of any URLLC packet at time t is upper bounded by*

(13) $$\begin{array}{cc} D(t) & \leq h\left(\alpha_{u}\left(t\right),\beta_{ch}\left(t\right)\right)=\frac{k}{R_{2}\left(n,P,\epsilon ,M\right)\cdot W},\\ Subject\;to:\; & \frac{k}{\tau }\leq R_{2}\left(n,P,\epsilon ,M\right)\cdot W. \end{array}$$

*where h(x(t),y(t)) is the maximum horizontal distance between functions of x(t) and y(t), defined as $h\left(x\left(t\right),y\left(t\right)\right)=\sup_{s\geq 0}\left\{\inf\{\tau \geq 0:x(s)\leq y(s+\tau )\}\right\}$.*

It could be decomposed that the delay is mainly determined by three parts: (1) how an information message is processed by coding and modulation scheme, indicated by codeword length *n* and modulation order *M*; (2) how the channel condition is when it is transmitted, indicated by SNR *P* and error probability ϵ; (3) how much system resource is allocated denoted by system bandwidth *W*. Therefore, optimization among these factors is needed, which are further discussed below.

### 3.3. Mcs Selection and Bandwidth Allocation

Usually, delay guarantee and error probability are required by specific URLLC service, the modulation and coding schemes are selected based on channel quality, which are all objective. Therefore, in order to provide strict delay and reliability guarantee for URLLC traffic, the system has to try it best to allocate reasonable amount of resource.

As it shows in Lemmas 1 and 2 that, the delay and error probability are strongly correlated with allocated spectrum *W*, coding scheme *n* and modulation order *M*. For any particular URLLC-tuple (k,d,ϵ), there are multiple solutions for (W,n,M). Here, we have Theorem 1 to reveal the relationship between MCS selection and bandwidth allocation, where the lower bound of allocated bandwidth is found.

**Theorem** **1.** *Consider an information packet with length of k bits going through a wireless channel under SNR of P, and the delay and error probability requirements are defined by $\left(d_{0},\epsilon_{0}\right)$, then, for a particular URLLC-tuple $\left(k,d_{0},\epsilon_{0}\right)$, there are multiple solutions for MCS selection $\left(n_{0},M_{0}\right)$ and bandwidth allocation $W_{0}$ as far as $W_{0}$ is no less than*

(14) $$W_{0}\geq \frac{k}{d_{0}\cdot R_{2}\left(P,n_{0},\epsilon_{0},M_{0}\right)}.$$

### 3.4. Effect of Data Duplication

In this section, we further extend the analysis to duplication transmission, which has been proposed as a fundamental enabler [9] for URLLC. The key concept of duplication transmission is to transmit the same information packet for several time either in time domain or in frequency domain. For time domain duplication, one URLLC information packet will be transmitted for several times in different time periods, which could improve the reliability but introduce extra delay, while for frequency domain duplication, several bandwidth resources will be used at the same time to transmit one URLLC information packet, which will improve the reliability without extra delay but occupy more spectrum resource. Note that hybrid duplication in both time domain and frequency domain is also possible. It is a tradeoff between time domain duplication and frequency domain duplication and will not be discussed in this work. The bandwidth allocation will be analyzed first, followed by discussions on average delay.

#### 3.4.1. Minimum Bandwidth Allcoation

When duplication transmission is applied, delay and error probability will be impacted relying on different duplication schemes. Then, the MCS selection and bandwidth allocation can be derived based on Theorem 1 as summarized in Corollary 1 followed by its proof.

**Corollary** **1.** *Consider a URLLC information packet with length of k bits, and it is transmitted for J times either consequently in time domain or parallelled in frequency domain under channel quality of P. If the overall delay and error probability after J times duplication is constraint by $\left(d_{0},\epsilon_{0}\right)$, there are multiple solutions for MCS selection $\left(n_{J},M_{J}\right)$ and bandwidth allocation $W_{J}$ as far as $W_{J}$ is no less than*

(15) $$W_{J}\geq J\cdot \frac{k}{d_{0}\cdot R_{2}\left(P,n_{J},\sqrt[{J}]{\epsilon_{0}},M_{J}\right)}.$$

**Proof.**  We first consider the duplication in time domain, which means the same information packet will be transmitted for *J* times consequently one by one using the same bandwidth. Since the maximum delay for URLLC and the length of information packet *k* are both short, we assume that the channel quality *P* as well as the coding and modulation scheme $\left(n_{J},M_{J}\right)$ will be the same for several duplicated transmissions. Let $\left(d_{t},\epsilon_{t}\right)$ denote the delay and error probability of each transmission, the worst case is that the last transmission is received correctly, therefore the maximum delay $d_{0}$ and overall error probability $\epsilon_{0}$ for *J* duplications in time domain will be

(16) $$\begin{array}{cc} d_{0} & =J\cdot d_{t},\\ \epsilon_{0} & =\epsilon_{0}^{J}. \end{array}$$

In order to fulfill the requirement of $\left(d_{0},\epsilon_{0}\right)$, the delay and error probability of each transmission $\left(d_{t},\epsilon_{t}\right)$ should be

(17) $$\begin{array}{cc} d_{t} & =d_{0}/J,\\ \epsilon_{t} & =\sqrt[{J}]{\epsilon_{0}}. \end{array}$$

By applying Theorem 1, the bandwidth allocation for each transmission $W_{t}$ should be no less than

(18) $$W_{t}\geq \frac{k}{d_{t}\cdot R_{2}\left(P,n_{J},\epsilon_{t},M_{J}\right)}=J\cdot \frac{k}{d_{0}\cdot R_{2}\left(P,n_{J},\sqrt[{J}]{\epsilon_{0}},M_{J}\right)}.$$

Since duplication is made in time domain, the bandwidth will be $W_{t}$ during *J* transmission.

When considering duplication in frequency domain, the same information packet will be transmitted for *J* times in different bandwidth resources at the same time. Let $\left(d_{f},\epsilon_{f}\right)$ denote the delay and error probability of each transmission, then the maximum delay $d_{0}$ and overall error probability $\epsilon_{0}$ for *J* duplications in frequency domain will be

(19) $$\begin{array}{c} d_{0}=d_{f},\\ \epsilon_{0}=\epsilon_{f}^{J}. \end{array}$$

The delay and error probability of each transmission $\left(d_{f},\epsilon_{f}\right)$ in time domain will be

(20) $$\begin{array}{cc} d_{f} & =d_{0},\\ \epsilon_{f} & =\sqrt[{J}]{\epsilon_{0}}. \end{array}$$

Based on Theorem 1, the bandwidth allocation for each transmission, denoted $W_{f}^{1}$, should be no less than

(21) $$W_{f}^{1}\geq \frac{k}{d_{f}\cdot R_{2}\left(P,n_{J},\epsilon_{f},M_{J}\right)}=\frac{k}{d_{0}\cdot R_{2}\left(P,n_{J},\sqrt[{J}]{\epsilon_{0}},M_{J}\right)}.$$

The whole bandwidth used for *J* duplications in frequency domain $W_{f}\left(J\right)$ will be

(22) $$W_{f}\geq J\cdot W_{f}^{1}=J\cdot \frac{k}{d_{0}\cdot R_{2}\left(P,n_{J},\sqrt[{J}]{\epsilon_{0}},M_{J}\right)}.$$

□

#### 3.4.2. Average Delay

By comparing Equations (18) and (22), it is proven that data duplication in time domain or in frequency domain requires the same spectrum allocation. However, the average delay are different. In time domain duplication, the transmission may be terminated when one transmission is received correctly, therefore, the average delay will be

(23) $$\begin{array}{c} \bar{D_{t}}=\sum_{i=1}^{i=J}i\cdot \frac{d_{0}}{J}\cdot \epsilon^{i-1}\left(1-\epsilon \right). \end{array}$$

In frequency domain duplication, all transmissions end at the same time, i.e., the average delay will be

(24) $$\begin{array}{c} \bar{D_{f}}=d_{0}. \end{array}$$

It is clear that the average delay of time domain duplication is smaller than the average delay of frequency domain duplication. However, in order to terminate the transmission in advance in time domain duplication, fast feedback mechanism is required to be implemented, which is out of the range of this work.

## 4. Evaluation Results

In 5G NR system, flexible configurations can be applied in order to fulfill different QoS requirements. 3GPP defines 32 MCS schemes to be used in Physical Downlink Shared CHannel (PDSCH) for 5G NR Rel.15 in Table 5.1.3.1-2 in [17]. In this work, the following 5 MCSs with significant different spectrum efficiency will be used in the evaluation as listed in Table 2. Note that, the coding rate defined in Table 5.1.3.1-2 in [17] is actually the binary code rate. For ease of understanding, the coding rate $r_{c}$ used in Equation (1) (named as overall code rate in Table 2) is also listed.

### 4.1. Snr Thresholds

**Figure 3:**
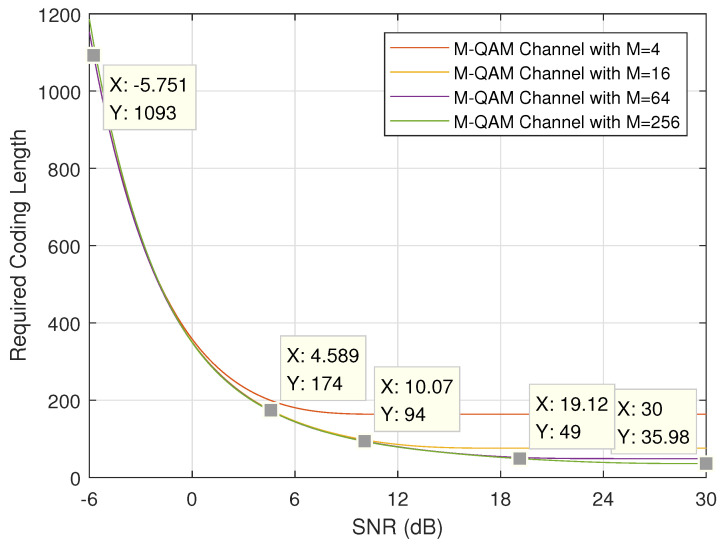
Required coding length with k=256 bits and $\epsilon =10^{-5}$.

**Figure 4:**
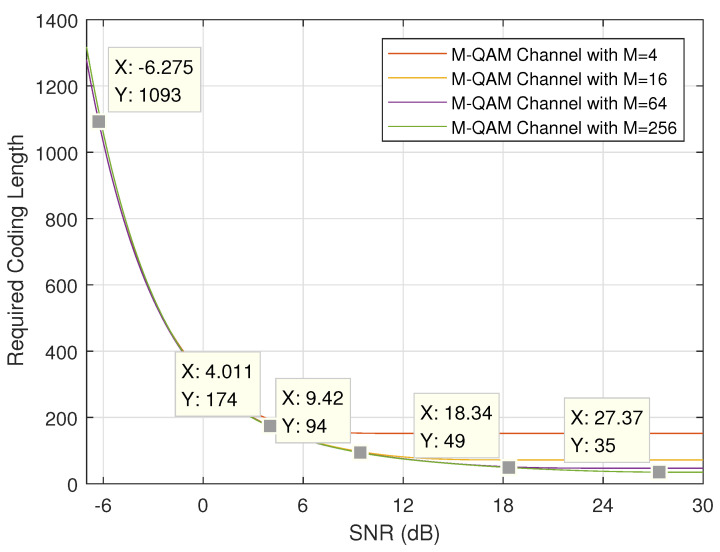
Required coding length with k=256 bits and $\epsilon =10^{-3}$.

First, we discuss the minimum required coding length under different SNRs. Figure 3 and Figure 4 show the theoretical results when the length of URLLC information bits *k* and error probability ϵ are set to $\left(256,\;10^{-5}\right)$ and $\left(256,\;10^{-3}\right)$, respectively. It is easy to notice that, when channel quality is good (i.e., SNR *P* is large) and modulation order is high (i.e., *M* is large), the required coding length will be short. These two figures plot the theoretical lower bound on coding length. When considering the practical MCS schemes defined in Table 2, the application feasibility and condition should be carefully discussed.

It is straightforward to calculate the practical coding lengths, denoted by $\hat{n}$ under different MCS indexes by applying Equation (1) when the information length is given. Table 3 lists the coding length when k=256 bits.

If we mark the practical coding length on the corresponding curve given in Figure 3 and Figure 4, the minimum required SNR can be found. To be specific, when MCS index 0 is applied, SNR should be no lower than −5.751 dB in order to guarantee error probability of $10^{-5}$ and SNR no lower than −6.275 dB in order to guarantee error probability of $10^{-3}$. Another important issue to be noted here is the point (X=30,Y=35.98), which means the required coding length is 35.98 when SNR is 30 dB. However, the practical coding length is only 35. In other words, the error probability of $10^{-5}$ cannot be guaranteed when applying MCS Index 27 even with high SNR of 30 dB (or even upto 50 dB which is not shown in this figure). For comparison, if error probability is lowered to $10^{-3}$, MCS Index 27 can be applied when SNR is higher than 27.37 dB as marked in Figure 4.

Based on the marks given in Figure 3 and Figure 4, SNR thresholds for adaptive MCS selection can be found as listed in Table 4, where the SNR thresholds for dual data duplication are also found. When the channel quality varies, suitable MCS scheme can be selected based on Table 4 dynamically in order to achieve better performance.

### 4.2. Impact of Error Probability

As can be seen from the SNR thresholds listed in Table 4, stronger reliability will require slightly higher SNR. This can be explained by observing the transmission rate given in Equation (7), which is re-written here as follows:

(25) $$R_{2}\left(n,P,\epsilon ,M\right)=I^{\prime }\left(P,M\right)-Loss\left(P,\epsilon ,n\right),$$

where

(26) $$Loss\left(P,\epsilon ,n\right)=\sqrt{\frac{V(P)}{n}}Q^{-1}\left(\epsilon \right)-\frac{\log_{2}\left(n\right)}{n}.$$

The second item Loss(P,ϵ,n) is composed of two parts and is introduced because of reliability requirement (denoted by ϵ) and finite coding length (denoted by *n*). Here, the capacity loss η(n,P,ϵ,M) is defined and discussed

(27) $$\eta \left(n,P,\epsilon ,M\right)=\frac{Loss(P,\epsilon ,n)}{I^{\prime }\left(P,M\right)}\times 100\%.$$

**Figure 5:**
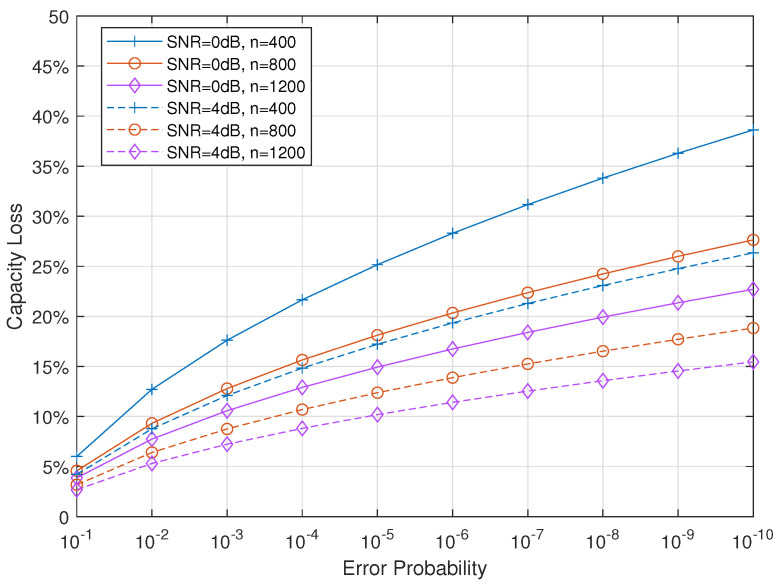
Capacity loss under different reliability requirements.

Figure 5 plots the capacity loss under different configurations, where the modulation order is set to 4. It can be seen that the loss increases when reliability requirement becomes stronger. However, the incremental rate of η is quite slow compared with the decreasing rate of ϵ.

### 4.3. Transmission Rate and Allocated Bandwidth

Given the allocated bandwidth, transmission rates under different MCS schemes can be obtained. In the frequency domain of NR system, a Resource Block (RB) is typically composed of 12 sub-carriers with interval of 15 kHz, which leads to a minimum scheduling unit of 180 kHz. Here, we use 540 kHz as the allocated bandwidth for URLLC traffic as an example.

**Figure 6:**
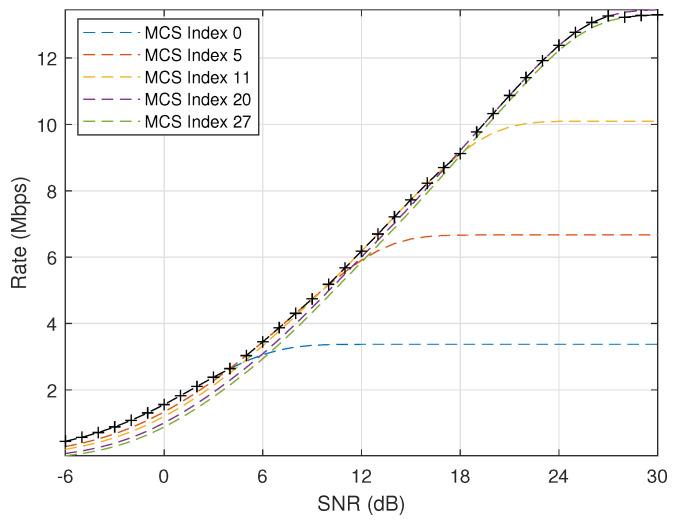
Transmission rate (k=256 bits, $\epsilon =10^{-3}$, W=540 kHz).

Figure 6 plots the theoretical transmission rate when k=256 bits and $\epsilon =10^{-3}$ under different MCS schemes. It is obvious that the transmission rate will be significantly improved when SNR increases or more efficient MCS scheme is applied. In addition, the curve with maker “+“ is composed of those transmission rates after considering adaptive MCS selection with SNR thresholds given in Table 4. It can be seen that optimal rates can be achieved after applying adaptive MCS selection.

**Figure 7:**
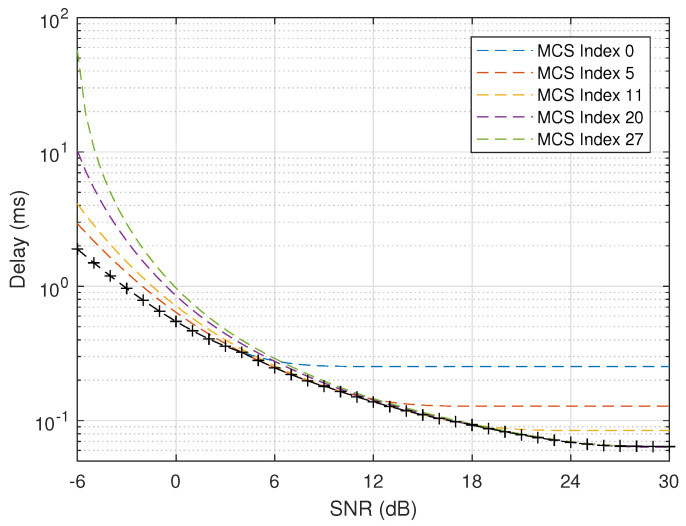
Maximum delay (k=256 bits, $\epsilon =10^{-3}$, W=540 kHz).

For the considered periodical URLLC traffic, the maximum theoretical delay can be calculated out by Equation (13) based on the transmission rate given in Figure 7.

In Figure 7, the maximum theoretical delay is more than 1 ms when SNR is below around −3 dB. If the periodical URLLC traffic can tolerate a maximum delay of 1 ms and error probability of $10^{-3}$, it means that more bandwidth should be allocated when SNR is below −3 dB, while less bandwidth can be allocated when SNR is above −3 dB in order to guarantee its QoS requirement and at the same time to consume as less system resource as possible.

**Figure 8:**
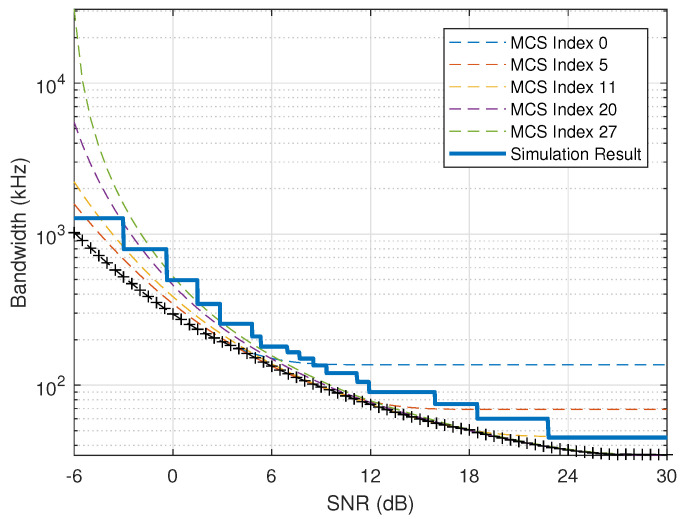
Capacity region (k=256 bits, $d_{0}=1$ ms, $\epsilon_{0}=10^{-3}$).

Based on Theorem 1 and Corollary 1, the minimum bandwidth derived theoretically under certain QoS requirement $\left(d_{0},\epsilon_{0}\right)$ and certain SNR can be obtained as shown by the slashed lines in Figure 8, where the QoS constraint is $(d_{0}=1$ ms, $\epsilon_{0}=10^{-3}$). The curves under fixed MCS schemes are ploted. In addition, the curve with marker + is composed of the minimum bandwidth among all MCS schemes considering adaptive MCS selection in different SNR ranges, and this curve can be used as admission region for QoS constraint of $(d_{0}=1$ ms, $\epsilon_{0}=10^{-3}$).

Simulation is also conducted to validate the theoretical analysis in this work. A simulation platform is established, where a typical deployment scenario is considered, i.e., an area of 19 cells (further divided into 57 sectors) with radius of 500 meters. Users with periodical URLLC traffic are uniformed deployed within the considered area, and user density is 10 users per sector, i.e., 570 users in total. All MCS schemes defined in [17] are used in the simulation, where the best suitable MCS scheme under different SNR will be selected in order to guarantee the QoS constraint of $(d_{0}=1$ ms, $\epsilon_{0}=10^{-3}$). The solid line in Figure 8 is formed of the simulation results.

Each step indicates one MCS scheme. It can be seen that the simulation results are slightly higher than the theoretical results, this is because the theoretical results are the lowest bound under ideal scenario and this also validates the theoretical analysis. In other words, the presented theoretical analysis framework can be relied on to obtain the admission region used for adaptive MCS selection and bandwidth allocation. Compared with simulation, theoretical model is more efficient and can be easily applied or extended to other scenarios.

## 5. Conclusions

In this work, we established a theoretical analysis model by considering all necessary factors during the transmission of a URLLC message, including the channel quality indicated by SNR, finite coding length, modulation scheme, delay and reliability requirements as well as information length and duplicated transmission. Network calculus was then applied to reveal the connection between the URLLC (k,d,ϵ)-tuple, modulation and coding scheme, channel quality and number of duplicated transmission, where the error probability, the maximum delay, the minimum bandwidth as well as the effect of duplicated transmissions were analyzed. The analysis helps to find the theoretical bounds, such as maximum delay given the allocated bandwidth and minimum required bandwidth given delay and reliability constraint. Configurations defined in the 5G NR network were used to obtain numerical results.

The adaptive MCS selection thresholds and admission region under certain delay and reliability constraint are presented and discussed. In addition, the theoretical analysis is validated by comparing with the simulation results yielded from the system-level simulation platform. The presented analysis framework can be extended to other scenarios, and the presented results for URLLC traffic can be applied by the network to conduct adaptive MCS selection and resource allocation.

## Figures and Tables

**Table 1:** Fitting coefficients for M-QAM.

*M*	$k_{M}$	$a_{1}^{\left(M\right)}$	$a_{2}^{\left(M\right)}$	$a_{3}^{\left(M\right)}$	$a_{4}^{\left(M\right)}$	$b_{1}^{\left(M\right)}$	$b_{2}^{\left(M\right)}$	$b_{3}^{\left(M\right)}$	$b_{4}^{\left(M\right)}$
256	4	0.228768	0.229083	0.118223	0.423927	0.183242	0.038011	0.994472	0.006911
64	4	0.198324	0.512831	0.209086	0.079759	0.408618	0.027517	0.120616	1.467118
16	3	0.658747	0.117219	0.224034	–	0.115521	1.467927	0.482023	–
4	2	0.143281	0.856719	–	–	1.557531	0.57239	–	–

**Table 2:** MCS configurations.

MCS	*M*	Binary	Overall Code
**Index**		**Code Rate**	**Rate** $r_{c}$
0	4	0.11719	0.2344
5	16	0.36914	1.4766
11	64	0.45508	2.7305
20	256	0.66650	5.3320
27	256	0.92578	7.4063

**Table 3:** Practical coding length in 5G NR (when k=256 bits).

MCS Index	Coding Length $\hat{n}$
0	1093
5	174
11	94
20	48
27	35

**Table 4:** SNR thresholds for MCS selection (when k=256 bits).

MCS Index	1	5	11	20	27
One time transmission ($\epsilon =10^{-5}$)	[−5.751, 4.589)	[4.589, 10.07)	[10.07, 19.12)	[19.12, ∞)	∕
One time transmission ($\epsilon =10^{-3}$)	[−6.275, 4.011)	[4.011, 9.42)	[9.42, 18.34)	[18.34, 27.37)	[27.37, ∞)
Dual duplication transmission $\left(\epsilon_{t}=\sqrt{10^{-5}}\right)$	[−6.442, 3.828)	[3.828, 9.192)	[9.192, 18.02)	[18.02, 25.98)	[25.98, ∞)
Dual duplication transmission $\left(\epsilon_{t}=\sqrt{10^{-3}}\right)$	[−6.841, 3.385)	[3.385, 8.673)	[8.673, 17.48)	[17.48, 24.72)	[24.72, ∞)

## Data Availability

Not applicable.
